# Lack of Association between Epidermal Growth Factor or Its Receptor and Reflux Esophagitis, Barrett's Esophagus, and Esophageal Adenocarcinoma: A Case-Control Study

**DOI:** 10.1155/2022/8790748

**Published:** 2022-08-31

**Authors:** Tereza Deissova, Michaela Cvanova, Zdenek Kala, Zuzana Jiraskova Zakostelska, Jiri Dolina, Lumir Kunovsky, Radek Kroupa, Zdenek Pavlovsky, Bretislav Lipovy, Zdenek Danek, Lydie Izakovicova Holla, Ondrej Urban, Vit Navratil, Robert Lischke, Tomas Harustiak, Tomas Grolich, Vladimir Prochazka, Ondrej Slaby, Petra Borilova Linhartova

**Affiliations:** ^1^RECETOX, Faculty of Science, Masaryk University, Kotlarska 2, 602 00 Brno, Czech Republic; ^2^Department of Pathophysiology, Faculty of Medicine, Masaryk University, Kamenice 735/5, 625 00 Brno, Czech Republic; ^3^Institute of Biostatistics and Analyses, Faculty of Medicine, Masaryk University, Kamenice 735/5, 625 00 Brno, Czech Republic; ^4^Department of Surgery, Institution Shared with University Hospital Brno, Faculty of Medicine, Masaryk University, Jihlavska 20, 625 00 Brno, Czech Republic; ^5^Institute of Microbiology of the Czech Academy of Sciences, Videnska 1083, 142 20 Prague, Czech Republic; ^6^Department of Gastroenterology and Internal Medicine, Institution Shared with University Hospital Brno, Faculty of Medicine, Masaryk University, Jihlavska 20, 625 00 Brno, Czech Republic; ^7^2nd Department of Internal Medicine - Gastroenterology and Geriatrics, University Hospital Olomouc, Faculty of Medicine and Dentistry, Palacky University, I. P. Pavlova 6, 779 00 Olomouc, Czech Republic; ^8^Department of Gastroenterology and Digestive Endoscopy, Masaryk Memorial Cancer Institute, Zluty Kopec 7, 656 53 Brno, Czech Republic; ^9^Department of Pathology, Institution Shared with University Hospital Brno, Faculty of Medicine, Masaryk University, Jihlavska 20, 625 00 Brno, Czech Republic; ^10^Department of Burns and Plastic Surgery, Institution Shared with University Hospital Brno, Faculty of Medicine, Masaryk University, Jihlavska 20, 625 00 Brno, Czech Republic; ^11^Clinic of Maxillofacial Surgery, Institution Shared with University Hospital Brno, Faculty of Medicine, Masaryk University, Jihlavska 20, 625 00 Brno, Czech Republic; ^12^Clinic of Stomatology, Institution Shared with St. Anne's University Hospital, Faculty of Medicine, Masaryk University, Pekarska 664/53, 602 00 Brno, Czech Republic; ^13^3rd Department of Surgery, First Faculty of Medicine, Charles University and Motol University Hospital, V Uvalu 84, 150 06 Prague, Czech Republic; ^14^Central European Institute of Technology, Masaryk University, Kamenice 735/5, 625 00 Brno, Czech Republic; ^15^Department of Medical Biology, Faculty of Medicine, Masaryk University, Kamenice 735/5, 625 00 Brno, Czech Republic

## Abstract

The epidermal growth factor (*EGF*) and its receptor (*EGFR*) gene-gene interactions were shown to increase the susceptibility to esophageal cancer. However, the role of the EGF/EGFR pathway in the development of gastroesophageal reflux disease (GERD) and its complications (reflux esophagitis (RE), Barrett's esophagus (BE), and esophageal adenocarcinoma (EAC)) remains unclear. This association study is aimed at investigating functional *EGF* and *EGFR* gene polymorphisms, their mRNA expression in esophageal tissues, and EGF plasma levels in relation to RE, BE, and EAC development in the Central European population. 301 patients with RE/BE/EAC (cases) as well as 98 patients with nonerosive reflux disease (NERD) and 8 healthy individuals (controls) were genotyped for +61 A>G *EGF* (rs4444903) and +142285 G>A *EGFR* (rs2227983) polymorphisms using the TaqMan quantitative polymerase chain reaction (qPCR). In random subgroups, the *EGF* and *EGFR* mRNA expressions were analyzed by reverse transcription qPCR in esophageal tissue with and without endoscopically visible pathological changes; and the EGF plasma levels were determined by enzyme-linked immunosorbent assay. None of the genotyped SNPs nor *EGF-EGFR* genotype interactions were associated with RE, BE, or EAC development (*p* > 0.05). Moreover, mRNA expression of neither *EGF* nor *EGFR* differed between samples of the esophageal tissue with and without endoscopically visible pathology (*p* > 0.05) nor between samples from patients with different diagnoses, i.e., RE, BE, or EAC (*p* > 0.05). Nevertheless, the lower *EGF* mRNA expression in carriers of combined genotypes AA +61 *EGF* (rs4444903) and GG +142285 *EGFR* (rs2227983; *p* < 0.05) suggests a possible direct/indirect effect of *EGF*-*EGFR* gene interactions on *EGF* gene expression. In conclusion, *EGF* and *EGFR* gene variants and their mRNA/protein expression were not associated with RE, BE or EAC development in the Central European population.

## 1. Introduction

Gastroesophageal reflux disease (GERD) is a common gastrointestinal illness developing when the reflux of gastric contents into the esophagus causes symptoms and/or complications—reflux esophagitis (RE), Barrett's esophagus (BE), and esophageal adenocarcinoma (EAC) [1, 2]. Patients with typical symptoms but no endoscopically visible esophageal mucosal injury are diagnosed with nonerosive reflux disease (NERD). Macroscopic mucosal lesions are visible in the RE, BE, and EAC. The progression from NERD to more severe forms of the disease or to GERD is uncommon [3, 4].

The epidermal growth factor (*EGF*) and its receptor (*EGFR*) signaling pathway plays an essential role not only during physiological maintenance of the epithelium (oral, nasal, esophageal, gastric, and intestinal mucosa) but also in numerous pathological processes (mucosal ulcers, inflammatory bowel diseases, etc.) [5–9]. A number of EGFR ligands cause allosteric changes in the intracellular domain of this transmembrane receptor and activation of tyrosine kinase [10]; of this receptor's ligands, the transforming growth factor *α* (TGF*α*) appears to play the most important role in the healing of acute mucosal defects, while EGF is predominantly involved in the healing of chronic ulcerations [6, 11, 12]. The biological function of the EGF/EGFR signaling pathway lies, in particular, in cell proliferation, migration, adhesion, and differentiation, as well as in the inhibition of gastric acid secretion (stimulation of the Na^+^/H^+^ exchanger) and in the protection of the mucosa from chemical, physical, and biological stresses [5, 13–15].

The effects of EGF on the healing of gastric or duodenal mucosa were demonstrated *in vivo* in rabbits and rats [16–19]. EGF is produced in many parts of the gastrointestinal tract (GIT), including salivary glands, pancreas, and Brunner's glands of the proximal duodenum. In addition, the application of exogenous EGF was shown to significantly increase the rate of wound healing in an EGFR-dependent manner in an *in vitro* model of vocal folds wound healing [20]. In addition, the inactivation of EGFR by deoxycholic acid activated an intestine-specific cascade typical for Barrett's metaplasia. Therefore, active EGFR signaling pathway may play a protective role in BE development [20, 21]. Conversely, the loss of this intestinal program and overactivation of EGFR lead to uncontrolled growth and progression from metaplasia to carcinoma [20, 22]. Moreover, in the process of premalignant progression of BE, the dysplastic BE cells and normal epithelial cells around them exhibit marked downregulation of the EGFR signaling pathway, which prevents neoplastic transformation [23].

**Figure 1:**
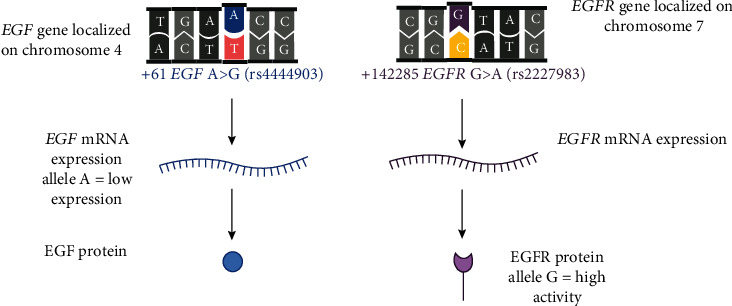
The function of studied polymorphisms based on previous studies; allele A of +61 A>G epidermal growth factor (rs4444903) polymorphism leads to a reduction of *EGF* mRNA expression, and allele G of +142285 G>A *EGF* receptor (*EGFR*, rs2227983) polymorphism increases the activity of EGFR [24, 25].

A functional polymorphism has been found in the *EGFR* gene; the variant +142285 G>A *EGFR* (rs2227983) is characterized by the amino acid substitution of arginine for lysine in the extracellular domain of the receptor and affects the affinity of EGFR ligands (EGF, TGF*α*), increases its tyrosine kinase activity, attenuates growth stimulation, and decreases the induction of protooncogenes Fos, Jun, and Myc [24]. Also, the expression of *EGF* could be affected by the +61 *EGF* A>G (rs4444903) functional polymorphism located in the 5′ untranslated (promoter) region of the *EGF* gene (see Figure 1) [25]. The G allele of +61 *EGF* A>G (rs4444903) polymorphism was associated with higher EGF serum levels in patients with GERD [26]. Moreover, the *EGF-EGFR* gene-gene interaction was shown to increase the susceptibility to esophageal cancer [27].

Based on previous findings in different populations [25–29], we aimed to find out if the variability in *EGF* and *EGFR* genes, their interaction, and expression constitute risk factors or disease markers of RE/BE/EAC development and progression in the Central European population. To this date, there is no study focused on *EGF/EGFR* gene variability in the European Caucasian population, and the findings from others cannot be reliably applied to this population due to interpopulational genetic differences. The presented study aimed to (1) analyze the two functional single-nucleotide polymorphisms (SNPs) in the *EGF* and *EGFR* genes and their gene-gene interactions in relation to the development of BE and EAC, (2) analyze *EGF* and *EGFR* mRNA expressions in the esophageal tissue samples with and without endoscopically visible pathological changes in GERD patients, and (3) compare EGF plasma levels in patients with GERD to those found in healthy controls from the Central European population.

## 2. Materials and Methods

### 2.1. Study Design, Inclusion and Exclusion Criteria, and Clinical and Histopathological Examination

The study was approved by the Ethics Committees of the Faculty of Medicine, Masaryk University (No. 09/2020, March 11^th^, 2020), University Hospital Brno (No. 01-290605/EK, June 29^th^, 2005, No. 05-101019/EK, May 15^th^, 2019), University Hospital Motol, Prague (without number, June 19^th^, 2019), and University Hospital Olomouc (No. 104/19, June 25^th^, 2019).

Written informed consent was obtained from all participants, in line with the Helsinki declaration, before inclusion in the study. In this study, a total of 407 individuals from the Czech and Slovak populations were enrolled. Subjects were examined at the Department of Gastroenterology, University Hospital Brno, Czech Republic, 3rd Department of Surgery, University Hospital Motol, Prague, Czech Republic, and Department of Gastroenterology and Geriatrics, University Hospital Olomouc, Czech Republic, between 2005 and 2021. Inclusion criteria were as follows: age ≥ 18 years, willingness to participate in the study and to sign the informed consent, and willingness to undergo endoscopic examination. Exclusion criteria were as follows: close family relationship to another participant in the study, other than Caucasian race, hepatic/renal failure, other types of tumors, and pregnancy.

All participants underwent esophagogastroduodenoscopy (EGD) with standard indications (patients with dyspepsia, dysphagia, epigastric pain, heartburn, regurgitation, anemia, etc.) and/or for this study purposes (individuals without digestive disease). During EGD, the duodenum, stomach, and esophagus were examined and biopsy samples taken. Patients were diagnosed endoscopically according to the Savary-Miller (SM) classification or Los Angeles classification; also, Barrett's C/M Prague criteria were used. BE was defined according to the European Society of Gastrointestinal Endoscopy guidelines [30]; EAC was confirmed by histological examination.

In this case-control association study, participants were divided into two groups according to their clinical diagnosis. Group 1 consisted of patients with endoscopically confirmed esophageal injury and/or complications related to GERD (RE, BE, and EAC). Patients with GERD-related symptoms but without visible pathology on endoscopy (NERD) and healthy individuals with respect to inclusion and exclusion criteria were included in Group 2. The flowchart of performed analyses is in the Supplementary Material (Figure S1).

### 2.2. Samples Collection, DNA, and RNA Isolation

From each subject, 9 mL of peripheral blood was collected into a tube containing 0.5 M EDTA (S-Monovette® 9 mL K3E, Sarstedt, Germany). Plasma was separated from these samples by centrifugation (2000 g, 4°C, 10 min) within 60 minutes of collection, aliquoted (6 × 300 *μ*L), and stored at −70°C until ELISA analysis. The remaining plasma was used for DNA isolation from leukocytes based on the modified salting-out method with proteinase K digestion of cells [31].

The biopsies from 23 patients with RE, BE, or EAC were collected only at the Department of Gastroenterology, University Hospital Brno, Czech Republic. Four biopsies were taken from each patient's esophagus during the endoscopic examination of the upper GIT. Two samples were collected from the part with endoscopically visible pathological changes and two from the part without such apparent changes. In this way, we acquired two pairs of samples, each pair containing one sample from the seemingly pathological and one from the seemingly healthy tissue. One pair was placed into 1.8 mL cryovials (SPL Life Sciences, Korea) with 1 mL of RNAlater™ Stabilization Solution (Thermo Fisher Scientific, Waltham, MA, USA) and stored at −70°C until RNA extraction. The other pair was sent to the Department of Pathology, Faculty Hospital Brno, Czech Republic, for histopathological confirmation of the diagnosis.

### 2.3. Genotyping of Polymorphisms in *EGF* and *EGFR*

This genetic association study comprised the entire study population (*n* = 407) and was designed as a case-control study. Genotyping of two functional SNPs +61 A>G *EGF* (rs4444903) and +142285 G>A *EGFR* (rs2227983) was performed by qPCR using 5′ nuclease TaqMan™ SNP Genotyping Assays (C__27031637_30 and C__16170352_20, respectively). The reaction mixture was prepared and conditions set in accordance with the manufacturer's instructions (Thermo Fisher Scientific, Waltham, MA, USA); fluorescence was measured using the Roche LightCycler® 96 System (Roche, Mannheim, Germany) at the Department of Pathophysiology, Faculty of Medicine, Masaryk University, Brno, Czech Republic. The LightCycler® 96 Application Software was used to analyze real-time and endpoint fluorescence data. Genotyping was verified by using positive control subjects in each 96-well plate and rerunning ≥ 5% of the samples, which were 100% concordant. The gene-gene interaction analysis was based on the method used by Upadhyay et al. [27] who modeled the combination of the genotypes bearing risk for GERD development, namely, +61 AA *EGF* (rs4444903) and+142285 GG *EGFR* (rs2227983).

### 2.4. Analysis of *EGF* and *EGFR* Gene Expressions

The relative quantifications of *EGF* and *EGFR* mRNA were performed in esophageal tissues with/without endoscopically visible pathological changes in 23 patients with GERD; namely, these comprised 10 patients with RE, 6 with BE, and 7 with EAC. Total RNA was isolated from fresh biopsies using AllPrep DNA/RNA/miRNA Universal Kit (Qiagen, Hilden, Germany). Firstly, the RNAlater™ Stabilization Solution (Thermo Fisher Scientific, Waltham, MA, USA) was removed. Subsequently, the tissues were homogenized 2 × 50 s at 6500 RPM in 600 *μ*L lysis buffer with 2 g of Ceramic Beads, 1.4 mm (Qiagen, Hilden, Germany) using Precellys® Evolution homogenizer (Bertin Technologies SAS, France). Isolated total RNA was quantified using the NanoDrop 2000c spectrophotometer (Thermo Fisher Scientific, Waltham, MA, USA) and stored at −70°C until use. The cDNA was transcripted using the Transcriptor first strand cDNA synthesis kit with a mix of random hexamer primers and an anchored-oligo(dT)18 primer. The reaction mixture and conditions were designed according to the manufacturer's instructions (Roche, Mannheim, Germany). Expression of target *EGF* or *EGFR* genes and housekeeping gene glyceraldehyde-3-phosphate dehydrogenase (*GAPDH*) was analyzed using the TaqMan™ Gene Expression Assays (Hs01099990_m1, Hs01076090_m1, and Hs02758991_g1). The manufacturer's procedure was followed (Thermo Fisher Scientific, Waltham, MA, USA), and fluorescence was measured using Roche LightCycler® 480 System (Roche, Mannheim, Germany) at the Department of Biochemistry, Faculty of Science, Masaryk University, Brno, Czech Republic. All reactions were performed in triplicates. The LightCycler® 480 Application Software was used to analyze the cycle threshold (Ct) values for relative gene quantification.

### 2.5. Analysis of EGF Plasma Levels

Plasma EGF levels were measured in 8 healthy individuals from Group 2 (healthy controls, HC) and 29 patients with GERD from Group 1 using the commercially available Human EGF, DuoSet® ELISA kit (Bio-Techne R&D Systems s.r.o., UK); namely, the 29 patients with GERD included 10 patients with RE, 9 with BE, and 10 with EAC, respectively. All tests were performed according to the manufacturers' recommendations.

### 2.6. Statistical Analysis

All statistical analyses were performed using the program IBM SPSS Statistics for Windows, version 26. The age distribution of the patients among groups was compared by Kruskal-Wallis or Mann–Whitney test. The genotype and allele frequencies, Hardy–Weinberg equilibrium (HWE), and differences in sex representation were tested using the Pearson *χ*^2^ test. As the patients differed in age and sex in the genetic association study, the results were adjusted for these parameters to be able to compare our results with those of the study by Upadhyay et al. [27] who also presented adjusted results. The results are supplemented with odds ratios (OR) and 95% confidence intervals (CI) from logistic regression analysis, where OR are related to all other genotypes. In the case of gene-gene interaction analysis, the ORs are related to the reference group. The reference genotype was established according to Upadhyay et al. [27] and compared with the rest of the genotypes in the group of GERD patients by logistic regression.

The variation in mRNA expressions in tissues with and without endoscopically visible pathological changes was evaluated using Wilcoxon signed ranked test. Kruskal-Wallis or Mann–Whitney tests were used to test the expression differences in tissues with and without endoscopically visible pathological changes in the groups of patients according to their diagnosis or studied polymorphism. The Kruskal-Wallis or Mann–Whitney tests were also performed to compare plasma concentrations among the groups. Graphs were created in the software OriginPro, Version 2021b (OriginLab Corporation, Northampton, MA, USA).

## 3. Results

### 3.1. Demographic Data of the Studied Population

The investigated population included 161 patients with RE, 92 with BE, and 48 with EAC, constituting Group 1 (*n* = 301). The 8 healthy individuals and 98 patients with NERD were included in Group 2. The demographic data are given for each analysis separately (see Table 1). Significant differences were found in the age distribution across groups in the populations used for the genetic association study (*p* < 0.001) and for the analysis of plasmatic EGF protein levels (*p* = 0.004, see Table 1). A post hoc analysis revealed that all pairs of groups in the genetic association study also differed significantly in age, except for the NERD vs RE (*p* > 0.05; data not shown); in the study of EGF plasma levels, none of the age differences in the individual groups were significant, with the exception of Group 2 (that consisted only of healthy individuals; median age 35.0) and patients with EAC (median age 68.0; *p* = 0.003; data not shown). The representation of men in the population used for the genetic association study was higher in Group 1 and its subgroups (RE, BE, EAC) than in Group 2 (*p* < 0.001; see Table 1). Where *EGF/EGFR* mRNA expression and EGF plasma levels analyses were concerned, the presence of men was similar among subgroups (*p* > 0.05; see Table 1).

### 3.2. Genetic Association Case-Control Study

A total of 407 individuals, including 301 patients with GERD and 106 persons in Group 2 (98 patients with NERD and 8 healthy control), were genotyped for +61 A>G *EGF* (rs4444903) and +142285 G>A *EGFR* (rs2227983) polymorphisms. The allele and genotype frequencies of neither of the two polymorphisms, adjusted for age and sex, differed between Group 1 and Group 2, even when comparing Group 2 to subgroups according to the specific diagnoses of RE, BE, or EAC, respectively (*p* > 0.05; see Table 2). Unadjusted data are shown in the Supplementary Material (Table S1).

In addition, none of the *EGF-EGFR* genotypes interactions showed effects on the risk of developing GERD or its complications in comparison with reference genotypes *EGF-EGFR* AG-AA, AG-AG, GG-AA, and GG-AG (*p* > 0.05; see Tables 3–4), adjusted for age and sex. Unadjusted data are shown in the Supplementary Material (Tables S2–S3).

### 3.3. Expression of *EGF* and *EGFR* Genes in the Esophageal Tissue

**Figure 2:**
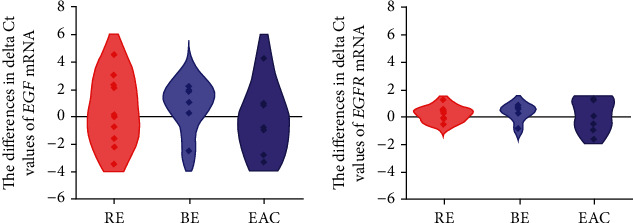
Violin plots of the differences in mRNA expression of the epidermal growth factor (*EGF*) and its receptor (*EGFR*) in the tissues with/without visible pathological changes among patients (*n* = 23) with reflux esophagitis (RE), Barrett's esophagus (BE) and esophageal adenocarcinoma (EAC). *p* > 0.05 Wilcoxon signed ranked test was used.

The expressions of *EGF*/*EGFR* mRNA were, according to delta-Ct values, similar in the esophageal tissues with/without endoscopically visible pathological changes in patients with GERD (*n* = 23; *p* > 0.05; data not shown). No differences in *EGF*/*EGFR* mRNA expressions were revealed among the RE, BE, and EAC tissue biopsies using the delta-delta Ct method, either (*p* > 0.05; Figure 2).

### 3.4. EGF Protein Levels in Plasma

**Figure 3:**
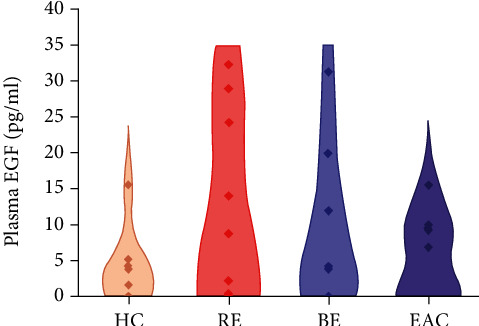
Violin plot of epidermal growth factor (EGF) plasma levels in 29 patients with gastroesophageal reflux disease (GERD), of which 10 suffered from reflux esophagitis (RE), 9 from Barrett's esophagus (BE), and 10 from esophageal adenocarcinoma (EAC), and 8 were healthy individuals (healthy controls, HC). *p* > 0.05; Wilcoxon signed ranked test was used.

Plasma levels of EGF did not significantly differ between patients with complications of GERD (RE, BE, or EAC) and healthy controls (HC; *p* >0.05; see Figure 3).

### 3.5. Relations between +61 A>G *EGF* (rs4444903) and +142285 *EGFR* G>A (rs2227983) Polymorphisms, EGF Plasma Levels, and *EGF* and *EGFR* mRNA Expressions in Esophageal Tissue

Our results showed that the polymorphism +61 A>G *EGF* (rs4444903) did not affect EGF plasma levels either in patients with GERD (*n* = 29) or in healthy controls (*n* = 8; *p* > 0.05; data not shown).

Relationships between genotypes and mRNA expressions were analyzed using the delta-delta Ct method. The *EGF* or *EGFR* mRNA expressions in esophageal tissue of GERD patients (*n* = 23) were independent on +61 A>G *EGF* (rs4444903) or +142285 G>A *EGFR* (rs2227983) polymorphisms (*p* > 0.05; data not shown).

**Figure 4:**
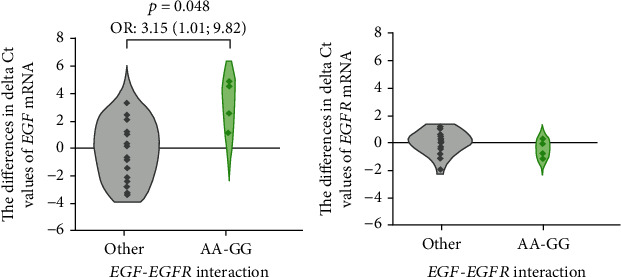
Violin plots of the differences in mRNA expression of the epidermal growth factor (*EGF)* and its receptor (*EGFR*) in the tissues with/without visible pathological changes from patients with gastroesophageal reflux disease (*n* = 23) divided into two groups according to their *EGF-EGFR* genotypes: +61 A>G *EGF* (rs4444903) and +142285 *EGFR* G>A (rs2227983) Kruskal-Wallis or Mann–Whitney tests were used.

However, the *EGF* mRNA expression was significantly lower in GERD patients with the genotype combination AA-GG (*EGF-EGFR*; *n* = 4, of which RE = 2 and EAC = 2) than in carriers of any other combination (*n* = 19; logistic regression: *p* = 0.048, OR: 3.15, see Figure 4).

## 4. Discussion

In our case-control study, we focused on a complex EGF/EGFR analysis in groups of patients with (Group 1) and without (Group 2) esophageal mucosal damage.

### 4.1. Genetic Association Case-Control Study

At first, we examined the functional +61 *EGF* A>G (rs4444903) and +142285 G>A *EGFR* (rs2227983) polymorphisms in GERD patients. These SNPs were analyzed by previous studies with controversial results. Lurje et al. associated the genotype AA of +61 *EGF* A>G (rs4444903) polymorphism with a higher likelihood of developing EAC recurrence [28]. Conversely, Lanuti et al. and Cheung et al. associated the presence of genotypes AG or GG of +61 *EGF* A>G (rs4444903) with an increased risk of EAC development in patients with GERD [26, 29]. In the case of the +142285 G>A *EGFR* (rs2227983) polymorphism, Yang et al. associated the allele A (phenotype with low activity of EGFR) with the risk of death and squamous cell carcinoma (ESCC) recurrence [32]. In addition, the *EGF-EGFR* interaction, especially the genotypes AA +61 *EGF* A>G (rs4444903; phenotype with low expression of *EGF*) and GG +142285 G>A *EGFR* (rs2227983), were shown to increase (2.5-fold) the susceptibility to esophageal cancer in a group of 159 patients with ESCC and 15 patients with EAC in comparison with a group of 196 endoscopically unexamined controls from the Indian population [27].

In our study, the +61 *EGF* A>G (rs4444903) and +142285 G>A *EGFR* (rs2227983) polymorphisms or their *EGF-EGFR* genotype interaction was not associated with the increased risk of GERD or its complications. In contrast to the study by Upadhyay et al. [27], our analysis was performed in 48 patients with EAC only vs. 106 endoscopically examined patients without inflammation or tissue changes in esophageal mucosa. The difference in results may be also affected by the interpopulational variability. According to NCBI, the minor allele frequencies (MAF) of +61 *EGF* A>G (rs4444903) and +142285 G>A *EGFR* (rs2227983) polymorphisms were 39.1% (allele G; rs4444903) and 27.6% (allele A; rs2227983), respectively, in the European (EUR) population compared with 54.1% (allele G; rs4444903) and 35.1% (allele A; rs2227983), respectively, in the South Asian (SAS) population. The MAFs in our “control” group corresponded to the EUR population (NCBI, *n* = 1006); specifically, the allele G of *EGF* rs4444903 was present in 41% (*p* = 0.586; *φ* = 0.012) of participants and the allele A of *EGFR* rs2227983 was carried by 24.5% (*p* = 0.335; *φ* = 0.020) of participants in our study. In addition, we analyzed not only distributions of studied polymorphisms, but also *EGF/EGFR* mRNA expression and EGF plasma level that were considered together with *EGF-EGFR* gene-gene interaction.

The main advantage of our study, compared with all others [25–29], lies in the fact that ours is the only one in which the control group consists of individuals with endoscopically and histopathologically examined esophagus. Limitations of our genetic association case-control study include the statistically significant differences in age and sex distributions across studied groups. These differences in our cohort were, nevertheless, expected because GERD progression is age-related [32, 33], and men are known to suffer from RE, BE, and EAC more frequently than women [34]. To eliminate this possible bias, the data were adjusted for both these parameters.

### 4.2. *EGF/EGFR* mRNA Expression Analysis

Even though it was reported that 90% of esophageal cancer show EGFR upregulation [35] and a recent meta-analysis found EGFR overexpression to be a predictive biomarker in clinical practice (because of its correlation with the clinicopathological features and overall survival prognostic value [36]), our study revealed no differences in *EGF* and *EGFR* mRNA expressions in esophageal tissues with or without endoscopically visible pathological changes in GERD patients. Moreover, we did not observe any changes in the *EGF* or *EGFR* mRNA expressions with the severity of the disease (RE, BE, or EAC).

Our results are consistent with the findings of a prospective study by Vallböhmer et al. who found no difference between *EGFR* mRNA expression in 59 patients with BE, dysplasia, or EAC (case group) and 16 patients with normal esophageal pH and no histological evidence of mucosal injury (control group). No correlation between *EGFR* mRNA expression and disease progression was detected in that study, either [37]. In addition, our results are in agreement with those recently reported by Wasielica-Berger et al. who found no significant changes in EGF or EGFR expression (examined by immunohistochemistry) in patients with erosive esophagitis compared to NERD patients. However, they revealed a positive correlation between EGFR expression and the presence of basal cell hyperplasia [38]. On the other hand, EGFR levels do not correlate with the EGFR signaling pathway activity that is mediated by an activation mutation or ligand binding. Baal et al. detected lower expression of phosphorylated (active) EGFR in BE tissues compared to the squamous esophageal tissue in the same patients (age range 44–86 years) [39]. However, it must be taken into account that the increased activation of EGFR could be associated with aging (as found in rats) [40]. In our *EGF/EGFR* expression analysis, the age and sex distributions were similar among subgroups (RE, BE, and EAC). The greatest strength of the presented study lies in the investigation of the *EGF/EGFR* expression in both types of tissues in the same GERD patients, which eliminates the effect of biological variability. Nevertheless, due to the relatively small number of patients, which remains a limitation of this part of the study, the results are rather indicative and should be verified in a larger cohort.

### 4.3. EGF Plasma Level Analysis

Finally, we analyzed the EGF plasma levels in patients with GERD. Benamouzig et al. did not find any association between the presence of RE with either EGFR expression or serum (or salivary) EGF levels [41]. In line with these findings, the EGF plasma levels were not associated with RE, BE, or with EAC in our patients. It seems more appropriate to study EGF levels in plasma than in the serum because, unlike EGF serum levels, EGF plasma levels are not correlated with the platelet count [42]. Also, in this case, it must be considered that the EGF blood levels change even naturally with age and sex. For example, the levels of EGF in platelet-rich plasma were shown to be higher in women than in men and in individuals younger than 26 years than in older ones, respectively [43]. EGF levels inversely correlate with age in healthy individuals [44]. In this part of our study, the sex distribution was similar among studied subgroups; however, the fact that the age was significantly different between Group 2 (consisting only of healthy individuals) and patients with EAC can be considered a limitation of this study.

### 4.4. Relations between +61 A>G *EGF* (rs4444903) and +142285 *EGFR* G>A (rs2227983) Polymorphisms and *EGF/EGFR* mRNA Expressions or EGF Plasma Levels

*EGF/EGFR* gene expression can be, besides transcription factors [45], miRNAs [46], hormones [47], and epigenetic modifications [48], also regulated by gene mutations. We assumed that the studied functional polymorphisms could influence EGF/EGFR production and, thus, contribute to disease progression. Lanuti et al. found out that the genotype GG of +61 *EGF* A>G (rs4444903) was significantly more common among the 312 patients with EAC than among 447 controls without a history of GERD (self-reported), in a mostly Caucasian population (98%). In addition, this GG genotype was associated with higher EGF serum levels in 82 patients with BE but not in those with GERD without endoscopically visible mucosal esophageal damages (*n* = 62) [26]. Unfortunately, it is not clear whether the higher EGF serum levels are associated with the presence of the GG genotype or with the presence of BE. Menke et al. reported a significantly increased frequency of the GG genotype of this SNP in patients with RE (*n* = 298), BE (*n* = 246), and EAC (*n* = 129) in comparison with endoscopically unexamined controls (*n* = 198) in a mostly Caucasian population. Moreover, the lower local EGF, investigated by immunohistochemical methods, was associated with carriage allele G of +61 *EGF* A>G (rs4444903) in 37 BE biopsies. Menke et al. suggested that the decreased EGF protein level in BE biopsies may support esophageal tumor development by reducing mucosal protection [49]. However, it is necessary to bear in mind that they examined the +61 *EGF* A>G (rs4444903) germinal variant in the genomic DNA from the samples of whole blood, not from BE biopsies, and the genotype in the affected tissue may differ from that observed in the whole blood.

In our study, the *EGF* mRNA expression in esophageal tissues or EGF plasma level was independent of the +61 *EGF* A>G (rs4444903) polymorphism. Similarly, the polymorphism +142285 G>A *EGFR* (rs2227983) was not associated with mRNA *EGFR* expression in the esophageal tissue of GERD patients. In contrast to a previous study using formalin-fixed, paraffin-embedded esophageal tissues, and immunohistochemistry for analysis of the EGF protein levels, we examined *EGF* mRNA expression in fresh esophageal tissues by RT-qPCR in our study. Moreover, we investigated *EGF* mRNA expression in both pathological and endoscopically normal esophageal tissues from the same patients to eliminate the biological variability. This could be one of the possible explanations for the observed differences in results. However, we found a significantly lower *EGF* mRNA expression in GERD patients with the combined AA-GG genotype (*EGF-EGFR*) that Upadhyay et al. [27] associated with the increasing risk of esophageal cancer. Our finding is in line with the study by Shahbazi et al. [25], where mononuclear cells from the peripheral blood of individuals with the AA genotype of +61 *EGF* A>G (rs4444903) produced significantly less *EGF* mRNA than the cells from the GG genotype carriers or heterozygous individuals. Also, Suenaga et al. associated the genotype AA of this SNP with lower tumoral *EGF* mRNA expression in Japanese patients with hepatocellular carcinoma [50]. It is possible that *EGF* expression can be directly or indirectly influenced by *EGF-EGFR* gene interaction. This relationship has not been described; hence, further analyses are needed for verification and explanation of these results on a larger sample.

## 5. Conclusions

The literature suggests that the EGF/EGFR signaling pathway plays a pleiotropic role in GERD development. While the active EGF/EGFR signaling pathway prevents the transformation of the normal esophageal squamous cell epithelium to BE, it also contributes to the malignant progression of BE. In our complex case-control study analysis, we have shown that neither (i) the +61 *EGF* A>G (rs4444903) and +142285 G>A *EGFR* (rs2227983) polymorphisms nor (ii) mRNA *EGF* or *EGFR* expressions and (iii) EGF plasma levels can be used as markers for the RE, BE, and EAC in the Central European population. In conclusion, our results show that the role of EGF/EGFR, especially functional gene variants, in BE and EAC development is not as important as we hypothesized.

However, the combination of genotypes AA-GG (*EGF-EGFR*) was associated with lower *EGF* mRNA expression; hence, the *EGF* mRNA expression may be directly or indirectly affected by the interaction of these genes.

## Figures and Tables

**Table 1:** Demographic data of subpopulations analyzed in individual partial analyses (genetic association study, *EGF/EGFR* mRNA expressions, and EGF plasma levels).

Analysis	Group 2^a^	RE	BE	EAC	*p* value^#^	Group 1^b^	*p* value^§^
*Genetic association study*							
Number (*n*)	106	161	92	48		301	
Age (median)	44.5	46.0	56.5	66.0	<0.001	53.0	<0.001
Sex (men, %)	55.7	72.0	81.5	75.0	<0.001	75.4	<0.001
*EGF/EGFR mRNA expression*							
Number (*n*)	—	10	6	7		23	
Age (median)	—	47.5	67.5	68.0	0.127	66.0	—
Sex (men, %)	—	90.0	66.7	85.7	0.644	82.6	—
*EGF plasma levels*							
Number (*n*)	8	10	9	10		29	
Age (median)	35.0	47.5	63.0	68.0	0.004	64.0	0.003
Sex (men, %)	50.0	90.0	66.7	70.0	0.306	75.9	0.203

BE: Barrett's esophagus; EAC: esophageal adenocarcinoma; GERD: gastroesophageal reflux disease group; NERD: nonerosive reflux disease group; RE: reflux esophagitis; Group 1: patients with diagnosis RE, BE, or EAC determined by a pathologist; Group 2: patients without macroscopical changes of the esophageal mucosa and with/without NERD (including healthy individuals); ^a^included 8 healthy individuals; ^b^included patients with RE, BE, and EAC; ^#^Group 2 vs. RE vs. BE vs. EAC comparison; ^§^Group 2 vs. Group 1 comparison.

**Table 2:** Allele and genotype frequencies of the +61 A>G *EGF* (rs4444903) and +142285 G>A *EGFR* (rs2227983) polymorphisms in study groups (*n* = 407), age- and sex-adjusted.

Diagnosis	Group 2 *n* = 106	RE *n* = 161	RE vs. Group 2 OR_adj_ (95% CI)	*p* value	BE *n* = 92	BE vs. Group 2 OR_adj_ (95% CI)	*p* value	EAC *n* = 48	EAC vs. Group 2 OR_adj_ (95% CI)	*p* value	Group 1 *n* = 301	Group 1 vs. Group 2 OR_adj_ (95% CI)	*p* value
*EGF A/G (rs4444903)*													
GG	16.0%	13.7%	0.85 (0.42-1.71)	0.642	21.7%	1.37 (0.60-3.09)	0.452	16.7%	1.79 (0.51-6.30)	0.366	16.6%	1.01 (0.53-1.91)	0.981
AG	50.0%	46.0%	0.86 (0.52-1.43)	0.567	38.0%	0.60 (0.31-1.14)	0.116	50.0%	0.80 (0.33-1.96)	0.627	44.2%	0.80 (0.50-1.27)	0.337
AA	34.0%	40.4%	1.28 (0.76-2.15)	0.354	40.2%	1.39 (0.73-2.67)	0.317	33.3%	0.93 (0.37-2.36)	0.879	39.2%	1.27 (0.78-2.07)	0.334
Allele G	41.0%	36.6%	0.85 (0.59-1.22)	0.373	40.8%	0.94 (0.59-1.48)	0.779	41.7%	1.20 (0.63-2.27)	0.577	38.7%	0.89 (0.64-1.25)	0.503
Allele A	59.0%	63.4%	1.18 (0.82-1.70)		59.2%	1.07 (0.68-1.68)		58.3%	0.83 (0.44-1.58)		61.3%	1.12 (0.80-1.57)	
*EGFR A/G (rs2227983)*													
AA	3.8%	7.5%	1.84 (0.57-5.96)	0.312	13.0%	3.04 (0.84-10.97)	0.089	8.3%	1.40 (0.25-7.93)	0.706	9.3%	2.22 (0.73-6.73)	0.157
AG	41.5%	38.5%	0.88 (0.53-1.46)	0.619	34.8%	0.70 (0.36-1.34)	0.277	37.5%	0.80 (0.32-1.98)	0.625	37.2%	0.84 (0.52-1.35)	0.464
GG	54.7%	54.0%	1.00 (0.61-1.66)	0.993	52.2%	1.03 (0.55-1.95)	0.920	54.2%	1.14 (0.47-2.78)	0.776	53.5%	0.99 (0.62-1.59)	0.978
Allele A	24.5%	26.7%	1.08 (0.72-1.63)	0.703	30.4%	1.19 (0.72-1.97)	0.495	27.1%	0.97 (0.48-1.98)	0.944	27.9%	1.13 (0.77-1.65)	0.537
Allele G	75.5%	73.3%	0.92 (0.62-1.39)		69.6%	0.84 (0.51-1.39)		72.9%	1.03 (0.51-2.08)		72.1%	0.89 (0.61-1.30)	

Adj: adjusted OR for age and sex; BE: Barrett's esophagus; CI: confidence interval; EAC: esophageal adenocarcinoma; EGF: epidermal growth factor; EGFR: epidermal growth factor receptor; NERD: nonerosive reflux disease group; OR: odds ratio; RE: reflux esophagitis; Group 1: patients with diagnosis RE, BE, or EAC determined by a pathologist; Group 2: patients without macroscopical changes of the esophageal mucosa and with/without NERD (including healthy individuals).

**Table 3:** Gene-gene interaction: +61 A>G *EGF* (rs4444903) and +142285 G>A *EGFR* (rs2227983) between study groups (*n* = 407), age- and sex-adjusted.

*EGF-EGFR* interaction	Group 2 *n* = 106		Group 1 *n* = 301		Group 1 vs. Group 2 OR_adj_ (95% CI)	*p* value
*EGF-EGFR* ^∗^	38	35.8%	91	30.2%	1.00 (ref.)	
AA-AA	3	2.8%	10	3.3%	1.44 (0.35-5.94)	0.614
AA-AG	7	6.6%	39	13.0%	2.43 (0.96-6.13)	0.060
AA-GG	26	24.5%	69	22.9%	1.17 (0.63-2.17)	0.625
AG-GG	25	23.6%	70	23.3%	1.25 (0.67-2.33)	0.489
GG-GG	7	6.6%	22	7.3%	1.41 (0.52-3.81)	0.497

Adj: adjusted OR for age and sex; CI: confidence interval; EGF: epidermal growth factor; EGFR: epidermal growth factor receptor; NERD: nonerosive reflux disease group; OR: odds ratio; ^∗^reference genotypes *EGF-EGFR* (AG-AA, AG-AG, GG-AA, and GG-AG) according to Upadhyay et al. [27]; Group 1: patients with diagnosis RE, BE, or EAC determined by a pathologist; Group 2: patients without macroscopical changes of the esophageal mucosa and with/without NERD (including healthy individuals).

**Table 4:** Gene-gene interaction: +61 A>G *EGF* (rs4444903) and +142285 G>A *EGFR* (rs2227983) between the NERD patients and individual subgroups with RE/BE/EAC (*n* = 407), age- and sex-adjusted.

*EGF-EGFR* interaction	Group 2 *n* = 106		RE *n* = 161		RE vs. Group 2 OR_adj_ (95% CI)	*p* value	BE *n* = 92		BE vs. Group 2 OR_adj_ (95% CI)	*p* value	EAC *n* = 48		EAC vs. Group 2 OR_adj_ (95% CI)	*p* value
*Reference* ^∗^	38	35.8%	49	30.4%	1.00 (ref.)		26	28.3%	1.00 (ref.)		16	33.3%	1.00 (ref.)	
AA-AA	3	2.8%	4	2.5%	0.98 (0.20-4.80)	0.983	5	5.4%	2.22 (0.40-12.38)	0.364	1	2.1%	0.57 (0.04-7.72)	0.676
AA-AG	7	6.6%	21	13.0%	2.31 (0.88-6.08)	0.091	13	14.1%	2.76 (0.83-9.15)	0.096	5	10.4%	3.01 (0.50-18.04)	0.228
AA-GG	26	24.5%	40	24.8%	1.19 (0.61-2.31)	0.604	19	20.7%	1.30 (0.55-3.07)	0.544	10	20.8%	0.99 (0.29-3.36)	0.988
AG-GG	25	23.6%	38	23.6%	1.22 (0.62-2.39)	0.562	19	20.7%	1.19 (0.49-2.88)	0.694	13	27.1%	1.48 (0.46-4.82)	0.514
GG-GG	7	6.6%	9	5.6%	1.11 (0.37-3.34)	0.859	10	10.9%	2.28 (0.65-7.93)	0.195	3	6.3%	2.86 (0.40-20.58)	0.295

Adj: adjusted OR for age and sex; BE: Barrett's esophagus; CI: confidence interval; EAC: esophageal adenocarcinoma; EGF: epidermal growth factor; EGFR: epidermal growth factor receptor; GERD: gastroesophageal reflux disease group; NERD: non-erosive reflux disease group; OR: odds ratio; RE: reflux esophagitis; ^∗^reference genotypes *EGF-EGFR* (AG-AA; AG-AG; GG-AA; GG-AG) according to Upadhyay et al. [27]; Group 2: patients without macroscopical changes of the esophageal mucosa and with/without NERD (including healthy individuals).

## Data Availability

The analyzed data used in this study are available from the corresponding author and the first author (raw data) upon request.

## Supplementary Materials

Supplementary 1 Figure S1: the flowchart of analyses performed in this study.

Supplementary 2 Table S1: allele and genotype frequencies of the +61 A>G *EGF* (rs4444903) and +142285 G>A *EGFR* (rs2227983) polymorphisms in study groups (*n* = 407).

Supplementary 3 Table S2: gene-gene interaction: +61 A>G *EGF* (rs4444903) and +142285 G>A *EGFR* (rs2227983) between study groups (*n* = 407).

Supplementary 4 Table S3: gene-gene interaction: +61 A>G *EGF* (rs4444903) and +142285 G>A *EGFR* (rs2227983) between the NERD patients and individual subgroups with RE/BE/EAC (*n* = 407).
