# Supplementary material for: Lack of Association between Epidermal Growth Factor or Its Receptor and Reflux Esophagitis, Barrett's Esophagus, and Esophageal Adenocarcinoma: A Case-Control Study
Source: Dis Markers. 2022 Aug 31;2022:8790748. doi: 10.1155/2022/8790748 (PMC9459439; doi:10.1155/2022/8790748)
Supplement: Supplementary 1 — Figure S1: the flowchart of analyses performed in this study. [file 8790748.f1.pptx]

## Slide 1
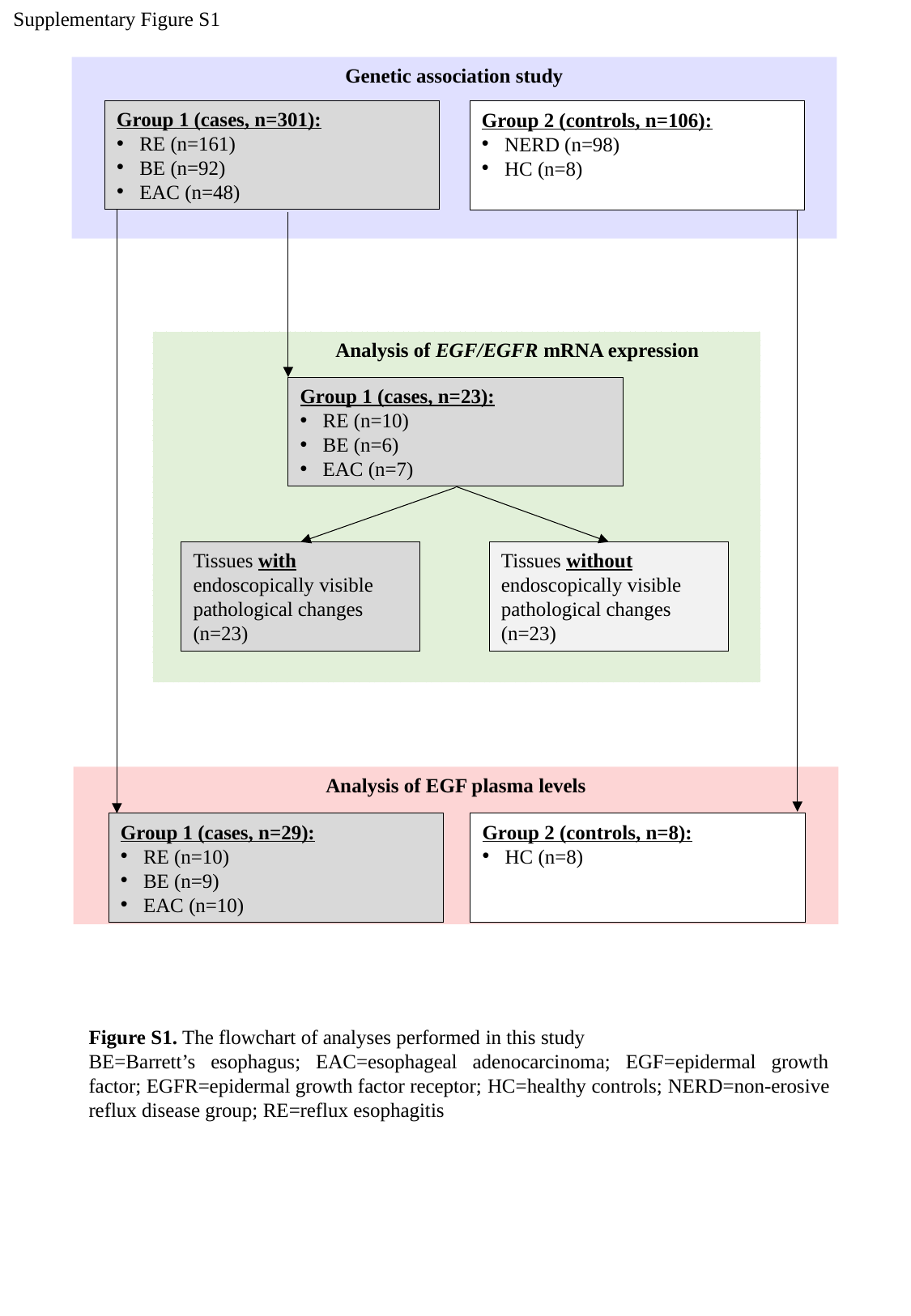

Supplementary Figure S1
Genetic association study
Group 1 (cases, n=301):
RE (n=161)
BE (n=92)
EAC (n=48)
Group 2 (controls, n=106):
NERD (n=98)
HC (n=8)
	Analysis of EGF/EGFR mRNA expression
Group 1 (cases, n=23):
RE (n=10)
BE (n=6)
EAC (n=7)
Tissues with endoscopically visible pathological changes (n=23)
Tissues without endoscopically visible pathological changes (n=23)
Analysis of EGF plasma levels
Group 1 (cases, n=29):
RE (n=10)
BE (n=9)
EAC (n=10)
Group 2 (controls, n=8):
HC (n=8)
Figure S1. The flowchart of analyses performed in this study
BE=Barrett’s esophagus; EAC=esophageal adenocarcinoma; EGF=epidermal growth factor; EGFR=epidermal growth factor receptor; HC=healthy controls; NERD=non-erosive reflux disease group; RE=reflux esophagitis
