# Supplementary material for: Lack of Association between Epidermal Growth Factor or Its Receptor and Reflux Esophagitis, Barrett's Esophagus, and Esophageal Adenocarcinoma: A Case-Control Study
Source: Dis Markers. 2022 Aug 31;2022:8790748. doi: 10.1155/2022/8790748 (PMC9459439; doi:10.1155/2022/8790748)
Supplement: Supplementary 2 — Table S1: allele and genotype frequencies of the +61 A>G EGF (rs4444903) and +142285 G>A EGFR (rs2227983) polymorphisms in study groups (n = 407). [file 8790748.f2.docx]

**Table S1**. Allele and genotype frequencies of the +61 A>G *EGF* (rs4444903) and +142285 G>A *EGFR* (rs2227983) polymorphisms in study groups (n=407)

| **Diagnosis** | **Group 2** n=106 | **RE** n=161 | **RE vs. Group 2** OR (95% CI) | ***p*-value** | **BE** n=92 | **BE vs. Group 2** OR (95% CI**)** | ***p*-value** | **EAC** n=48 | **EAC vs. Group 2** OR (95% CI) | ***p*-value** | **Group 1** n=301 | **Group 1 vs. Group 2** OR (95% CI) | ***p*-value** |
| --- | --- | --- | --- | --- | --- | --- | --- | --- | --- | --- | --- | --- | --- |
| ***EGF* A/G (rs4444903)** | | | |  |  |  |  |  |  |  |  |  |  |
| **GG** | 16.0% | 13.7% | 0.83 (0.42-1.65) | 0.591 | 21.7% | 1.45 (0.71-2.98) | 0.306 | 16.7% | 1.05 (0.42-2.63) | 0.922 | 16.6% | 1.04 (0.57-1.90) | 0.891 |
| **AG** | 50.0% | 46.0% | 0.85 (0.52-1.39) | 0.518 | 38.0% | 0.61 (0.35-1.08) | 0.092 | 50.0% | 1.00 (0.51-1.98) | 1.000 | 44.2% | 0.79 (0.51-1.23) | 0.302 |
| **AA** | 34.0% | 40.4% | 1.32 (0.79-2.19) | 0.291 | 40.2% | 1.31 (0.73-2.33) | 0.363 | 33.3% | 0.97 (0.47-2.00) | 0.939 | 39.2% | 1.25 (0.79-1.99) | 0.339 |
|  |  |  |  |  |  |  |  |  |  |  |  |  |  |
| **allele G** | 41.0% | 36.6% | 0.83 (0.58-1.19) | 0.307 | 40.8% | 0.99 (0.66-1.48) | 0.955 | 41.7% | 1.03 (0.63-1.67) | 0.917 | 38.7% | 0.91 (0.66-1.25) | 0.550 |
| **allele A** | 59.0% | 63.4% | 1.20 (0.84-1.72) |  | 59.2% | 1.01 (0.68-1.51) |  | 58.3% | 0.97 (0.60-1.59) |  | 61.3% | 1.10 (0.80-1.52) |  |
| ***EGFR* A/G (rs2227983)** | | | |  |  |  |  |  |  |  |  |  |  |
| **AA** | 3.8% | 7.5% | 2.05 (0.64-6.55) | 0.224 | 13.0% | 3.82 (1.19-12.31) | 0.024 | 8.3% | 2.32 (0.55-9.69) | 0.249 | 9.3% | 2.62 (0.90-7.64) | 0.079 |
| **AG** | 41.5% | 38.5% | 0.88 (0.54-1.45) | 0.624 | 34.8% | 0.75 (0.42-1.34) | 0.332 | 37.5% | 0.85 (0.42-1.70) | 0.639 | 37.2% | 0.84 (0.53-1.31) | 0.434 |
| **GG** | 54.7% | 54.0% | 0.97 (0.59-1.59) | 0.913 | 52.2% | 0.90 (0.52-1.58) | 0.720 | 54.2% | 0.98 (0.49-1.94) | 0.949 | 53.5% | 0.95 (0.61-1.48) | 0.827 |
|  |  |  |  |  |  |  |  |  |  |  |  |  |  |
| **allele A** | 24.5% | 26.7% | 1.12 (0.75-1.67) | 0.574 | 30.4% | 1.35 (0.86-2.10) | 0.189 | 27.1% | 1.14 (0.66-1.98) | 0.633 | 27.9% | 1.19 (0.83-1.71) | 0.341 |
| **allele G** | 75.5% | 73.3% | 0.89 (0.60-1.33) |  | 69.6% | 0.74 (0.48-1.16) |  | 72.9% | 0.88 (0.51-1.51) |  | 72.1% | 0.84 (0.59-1.20) |  |

BE=Barrett’s esophagus; CI=confidence interval; EAC=esophageal adenocarcinoma; EGF=epidermal growth factor; EGFR=epidermal growth factor receptor; NERD=non-erosive reflux disease group; OR=odds ratio; RE=reflux esophagitis

Group 1 = patients with diagnosis RE, BE, or EAC determined by a pathologist

Group 2 = patients without macroscopical changes of the esophageal mucosa and with/without NERD (including healthy individuals)
