# Supplementary material for: Lack of Association between Epidermal Growth Factor or Its Receptor and Reflux Esophagitis, Barrett's Esophagus, and Esophageal Adenocarcinoma: A Case-Control Study
Source: Dis Markers. 2022 Aug 31;2022:8790748. doi: 10.1155/2022/8790748 (PMC9459439; doi:10.1155/2022/8790748)
Supplement: Supplementary 3 — Table S2: gene-gene interaction: +61 A>G EGF (rs4444903) and +142285 G>A EGFR (rs2227983) between study groups (n = 407). [file 8790748.f3.docx]

**Table S2.** Gene-gene interaction: +61 A>G *EGF* (rs4444903) and +142285 G>A *EGFR* (rs2227983) between study groups (n=407)

| ***EGF-EGFR* interaction** | **Group 2**  n=106 | | **Group 1**  n=301 | | **Group 1 vs. Group 2**  OR (95% CI) | ***p*-value** |
| --- | --- | --- | --- | --- | --- | --- |
| ***EGF-EGFR***^*^ | 38 | 35.8% | 91 | 30.2% | 1.00 (ref.) |  |
| **AA-AA** | 3 | 2.8% | 10 | 3.3% | 1.39 (0.36-5.34) | 0.630 |
| **AA-AG** | 7 | 6.6% | 39 | 13.0% | 2.33 (0.96-5.66) | 0.063 |
| **AA-GG** | 26 | 24.5% | 69 | 22.9% | 1.11 (0.62-2.00) | 0.732 |
| **AG-GG** | 25 | 23.6% | 70 | 23.3% | 1.17 (0.65-2.12) | 0.605 |
| **GG-GG** | 7 | 6.6% | 22 | 7.3% | 1.31 (0.52-3.33) | 0.567 |

CI=confidence interval; EGF=epidermal growth factor; EGFR=epidermal growth factor receptor; NERD=non-erosive reflux disease group; OR=odds ratio

^*^reference genotypes *EGF-EGFR* (AG-AA; AG-AG; GG-AA; GG-AG) according to Upadhyay *et al*. [28]

Group 1 = patients with diagnosis RE, BE, or EAC determined by a pathologist

Group 2 = patients without macroscopical changes of the esophageal mucosa and with/without NERD (including healthy individuals)
