# Supplementary material for: Lack of Association between Epidermal Growth Factor or Its Receptor and Reflux Esophagitis, Barrett's Esophagus, and Esophageal Adenocarcinoma: A Case-Control Study
Source: Dis Markers. 2022 Aug 31;2022:8790748. doi: 10.1155/2022/8790748 (PMC9459439; doi:10.1155/2022/8790748)
Supplement: Supplementary 4 — Table S3: gene-gene interaction: +61 A>G EGF (rs4444903) and +142285 G>A EGFR (rs2227983) between the NERD patients and individual subgroups with RE/BE/EAC (n = 407). [file 8790748.f4.docx]

**Table S3.** Gene-gene interaction: +61 A>G *EGF* (rs4444903) and +142285 G>A *EGFR* (rs2227983) between the NERD patients and individual sub-groups with RE/BE/EAC (n=407)

| ***EGF-EGFR***  **interaction** | **Group 2**  n=106 | | **RE** n=161 | | **RE vs. Group 2** OR (95% CI) | ***p*-value** | **BE** n=92 | | **BE vs. Group 2** OR (95% CI) | ***p*-value** | **EAC** n=48 | | **EAC vs. Group 2** OR (95% CI) | ***p*-value** |
| --- | --- | --- | --- | --- | --- | --- | --- | --- | --- | --- | --- | --- | --- | --- |
| **reference**^*^ | 38 | 35.8% | 49 | 30.4% | 1.00 (ref.) |  | 26 | 28.3% | 1.00 (ref.) |  | 16 | 33.3% | 1.00 (ref.) |  |
| **AA-AA** | 3 | 2.8% | 4 | 2.5% | 1.03 (0.22-4.90) | 0.966 | 5 | 5.4% | 2.44 (0.54-11.09) | 0.250 | 1 | 2.1% | 0.79 (0.08-8.20) | 0.845 |
| **AA-AG** | 7 | 6.6% | 21 | 13.0% | 2.33 (0.90-6.04) | 0.083 | 13 | 14.1% | 2.71 (0.95-7.72) | 0.061 | 5 | 10.4% | 1.70 (0.47-6.15) | 0.421 |
| **AA-GG** | 26 | 24.5% | 40 | 24.8% | 1.19 (0.62-2.29) | 0.595 | 19 | 20.7% | 1.07 (0.49-2.32) | 0.868 | 10 | 20.8% | 0.91 (0.36-2.33) | 0.849 |
| **AG-GG** | 25 | 23.6% | 38 | 23.6% | 1.18 (0.61-2.28) | 0.625 | 19 | 20.7% | 1.11 (0.51-2.42) | 0.791 | 13 | 27.1% | 1.24 (0.51-3.00) | 0.642 |
| **GG-GG** | 7 | 6.6% | 9 | 5.6% | 1.00 (0.34-2.92) | 0.996 | 10 | 10.9% | 2.09 (0.70-6.19) | 0.184 | 3 | 6.3% | 1.02 (0.23-4.44) | 0.981 |

BE=Barrett’s esophagus; CI=confidence interval; EAC=esophageal adenocarcinoma; EGF=epidermal growth factor; EGFR=epidermal growth factor receptor; NERD=non-erosive reflux disease group; OR=odds ratio; RE=reflux esophagitis

^*^reference genotypes *EGF-EGFR* (AG-AA; AG-AG; GG-AA; GG-AG) according to Upadhyay *et al*. [28]

Group 2 = patients without macroscopical changes of the esophageal mucosa and with/without NERD (including healthy individuals)
